# Impact of SumiLarv^®^ 2MR on *Aedes aegypti* larvae: a multicenter study in Brazil

**DOI:** 10.1186/s13071-023-06064-w

**Published:** 2024-02-26

**Authors:** Josiane Nogueira Müller, Allan Kardec Ribeiro Galardo, Ana Paula Sales de Andrade Corrêa, Maria de Lourdes da Graça Macoris, Maria Alice Varjal de Melo-Santos, Mitsue Maia Nakazawa, Ademir Jesus Martins, José Bento Pereira Lima

**Affiliations:** 1 Laboratory of Biology, Control and Surveillance of Vector Insects—LBCVIV FIOCRUZ/RJ, Rio de Janeiro, Brazil; 2 Laboratory of Medical Entomology, Institute of Scientific and Technological Research of the State of Amapá—IEPA, Macapá, Brazil; 3 Programa de Pós-graduação em Medicina Tropical, Oswaldo Cruz Institute, FIOCRUZ, Rio de Janeiro, Brazil; 4 Superintendence for Endemic Disease Control, SUCEN, Marília, São Paulo State Brazil; 5 Entomology Department, Aggeu Magalhães Institute —FIOCRUZ/PE, Recife, Brazil

**Keywords:** Pyriproxyfen, Mosquito, Vector control

## Abstract

**Background:**

*Aedes aegypti* is associated with dengue, Zika, and chikungunya transmission. These arboviruses are responsible for national outbreaks with severe public health implications. Vector control is one of the tools used to prevent mosquito proliferation, and SumiLarv^®^ 2MR is an alternative commercial product based on pyriproxyfen for larval/pupal control. In this study, the residual effectiveness of SumiLarv^®^ 2MR in different regions of Brazil was evaluated in simulated field conditions.

**Methods:**

We conducted a multicenter study across four Brazilian states—Amapá, Pernambuco, Rio de Janeiro, and São Paulo—given the importance to the country’s climatic variances in the north, northeast, and southeast regions and their influence on product efficiency. The populations of *Ae. aegypti* from each location were held in an insectary. Third-instar larvae (L3) were added every 2 weeks to water containers with SumiLarv^®^ 2MR discs in 250-, 500- and 1000-l containers in Amapá and Rio de Janeiro, and 100-l containers in Pernambuco and São Paulo, using concentrations of 0.04, 0.08, and 0.16 mg/l.

**Results:**

Adult emergence inhibition over 420 days was observed in all tests conducted at a concentration of 0.16 mg/l; inhibition for 308–420 days was observed for 0.08 mg/l, and 224–420 days for 0.04 mg/l.

**Conclusions:**

Sumilarv^®^ 2MR residual activity demonstrated in this study suggests that this new pyriproxyfen formulation is a promising alternative for *Aedes* control, regardless of climatic variations and ideal concentration, since the SumiLarv^®^ 2MR showed adult emergence inhibition of over 80% and residual activity greater than 6 months, a period longer than that recommended by the Ministry of Health of Brazil between product re-application in larval breeding sites.

**Graphical abstract:**

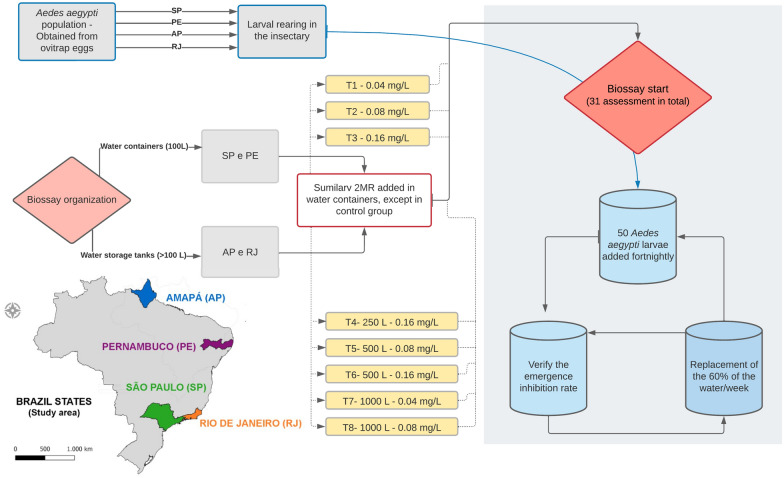

**Supplementary Information:**

The online version contains supplementary material available at 10.1186/s13071-023-06064-w.

## Background

The control of *Aedes aegypti* remains a public health challenge worldwide, especially in countries like Brazil, where socio-environmental factors such as the lack of basic sanitation are common among various municipalities, with open sewer drains and water storage containers. The use of water in these containers is typically intense, leading to loss of the larvicide applied for control of the *Ae. aegypti* population. In this context, new products or approaches for vector control are necessary to reduce the mosquito population in areas with transmission of dengue virus (DENV), Zika virus (ZIKV, and chikungunya virus (CHIKV) [1, 2].

Integrated vector management (IVM) is increasingly recommended by the World Health Organization (WHO) as a rational decision-making process that seeks the best use of resources for efficacy, cost-effectiveness, and sustainability of vector-borne disease control [3]. Different strategies addressing larvicides such as *Bacillus thuringiensis israelensis* (Bti), spinosad, and pyriproxyfen (PPF) are being evaluated [4–7] as a part of the Brazilian Dengue Control Program (PNCD), since resistance to temephos has been detected in some Brazilian field populations of *Ae. aegypti* [8, 9].

Pyriproxyfen is an insect growth regulator (IGR) and an analogue of the juvenile hormone used to inhibit the metamorphosis of mosquito larva, preventing its normal development into adulthood. SumiLarv 2MR is a novel product based on PPF formulated as a small resin disc with slow release of PPF through the matrix, designed to provide long persistence of lethal activity [10] in breeding sites. Studies have shown successful control using SumiLarv^®^ 2MR in Asia associated with the communication for behavioral impact (COMBI) program [11, 12].

The main objective of this study was to evaluate the residual effectiveness of different concentrations of SumiLarv^®^ 2MR against four populations of *Ae. aegypti* mosquitoes in different Brazilian regions. This was the first multicenter collaborative study to use this formulation in Brazil, and it aims to demonstrate the different uses, strengths, and limitations of the novel product in vector control of *Aedes*, which could help decision-makers to improve the *Aedes* control program in the country.

## Methods

The semi-field test (bioassay) was conducted in the states of Amapá, Rio de Janeiro, Pernambuco, and São Paulo. In Amapá, the study was performed in the city of Macapa at the Institute for Scientific and Technological Research of the State of Amapá (IEPA). In Rio de Janeiro, it was held in the capital of the state, Rio de Janeiro city, at the Laboratory of Biology, Control and Surveillance of Vector Insects (LBCVIV, Oswaldo Cruz Institute [IOC]/Oswaldo Cruz Foundation [Fiocruz]). In Pernambuco, the study took place at the Department of Entomology, Aggeu Magalhães Institute (IAM)/Fiocruz, in the capital city Recife. In São Paulo, the research was carried out in Marília, a city in the countryside of the state, at the Superintendency for the Control of Endemic Diseases (SUCEN). The bioassays were placed in an outdoor area, conducted in a shaded or partially shaded area, without direct sun exposure.

### *Aedes aegypti* population

Eggs were obtained from the *Ae. aegypti* females using ovitraps [13] in each city where the study was conducted to build the parental generation and preserve the local background. The larvae from the first offspring (F1) generation used in the test were reared in a synchronized way to avoid variations between them. The maintenance (feeding and density) of the larval rearing followed the protocols developed by researchers [14, 15], with a standard feeding ratio of 1 mg/larva.

### SumiLarv^®^ 2MR

SumiLarv^®^ 2MR is a product supplied by Sumitomo Chemical Company and recommended by WHO as the first long-lasting resin-based larvicide for treatment of breeding sites of mosquitoes, such as water containers [10]. The active ingredient/synergist is PPF at a concentration of 2% and radius of 5 cm. The dosage is 1 disc/40–500 l water, depending on local registration, with a duration of up to 6 months.

### Semi-field test (bioassay)

**Fig. 1:**
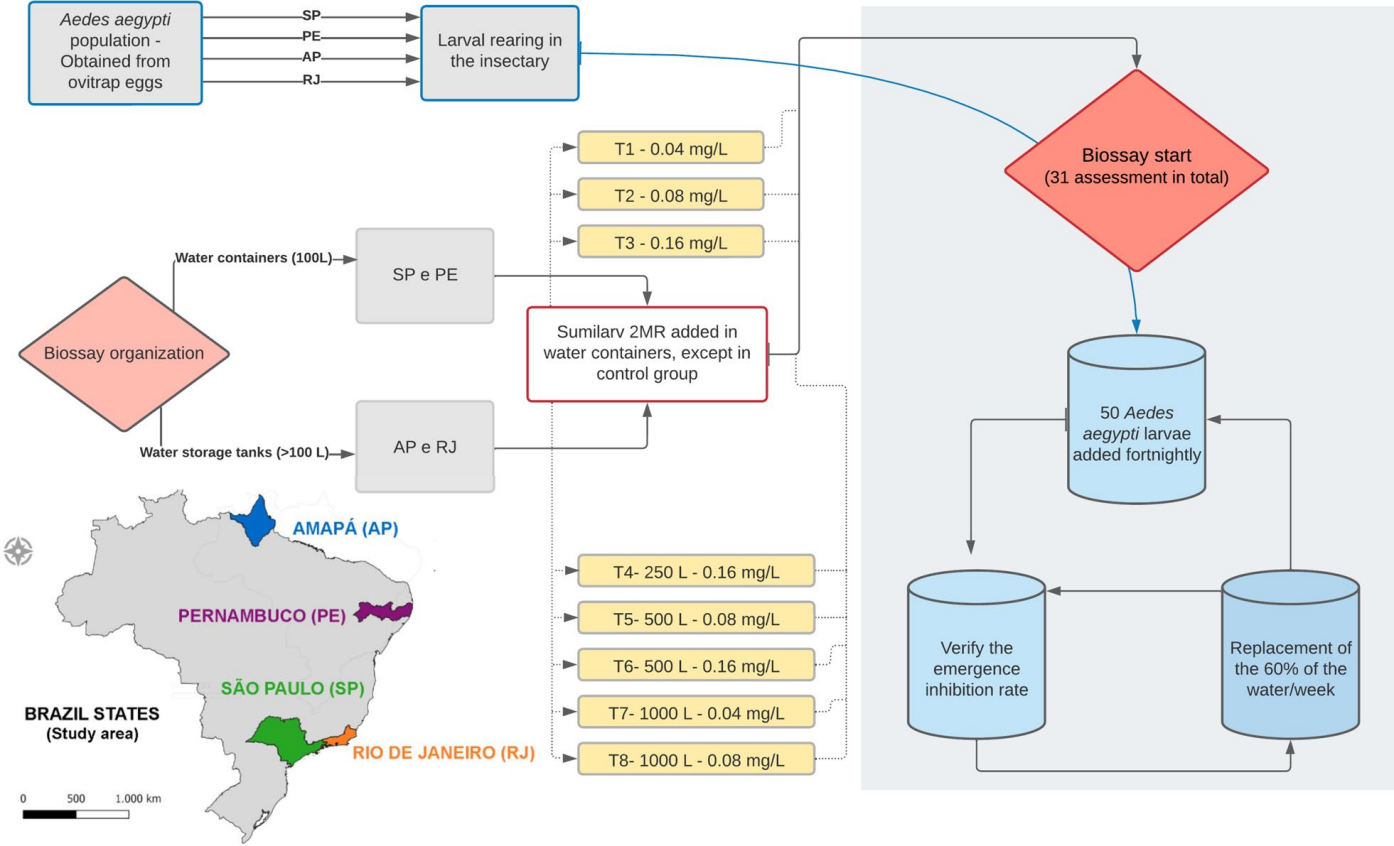
Multicenter bioassay with eight tests using SumiLarv^®^ 2MR in water containers in different concentrations. Period of the study: 2017–2019. Brazilian states: *PE* Pernambuco, *SP* São Paulo, *AP* Amapá, *RJ* Rio de Janeiro. *T* test

This study evaluated the residual larvicidal activity in a bioassay that was conducted in eight semi-field experiments in four different Brazilian regions. In Pernambuco and São Paulo, 100 l of water was used for the bioassays in the following concentrations: 0.04 (test 1), 0.08 (test 2), and 0.16 mg/l (test 3) (Fig. 1); the disks were cut to sizes appropriate for the volume of each container, and then weighed on a high-precision electronic scale. In Amapá and Rio de Janeiro, the whole disc was placed in water storage tanks of 250 (test 4), 500 (tests 5 and 6), and 1000 l (tests 7 and 8), proportionally maintaining the concentrations as shown in Fig. 1. Sixty percent of the water was replaced weekly, and each test consisted of three treated containers and one control group.

Three days before use, all containers were washed to remove possible plastic residues and then filled with water to confirm their integrity. This procedure was also carried out for the dechlorination of water obtained from the public supply system of each city, except in Amapá, where well water was used.

After the addition of 50 third-instar larvae (L3), the containers were treated with the SumiLarv^®^ 2MR product (the day of treatment day considered day 0). This procedure was repeated biweekly, adding the same number of *Ae. aegypti* larvae per replicate [15]. The protocol to verify the emergence inhibition rate consisted of a first assessment 72 h after the introduction of the larvae, followed by assessments every 48 h until all adults from the control group had emerged. Climate data (temperature and pH) in the water containers were recorded to analyze the environmental characteristics of each region where the bioassays were conducted. A pH meter/thermometer (K39-0014PA, KASVI) was used in the morning period. The assays began in November 2017 and lasted until March 2019. The criterion for completing the test was an emergence inhibition rate < 80% after 31 assessments or for two consecutive biweekly assessments.

### Data analysis

Emergence inhibition rate results were compared between mosquito populations from São Paulo and Pernambuco (tests 1–3), and between Amapá and Rio de Janeiro (tests 4–8). The exclusion criterion for the biweekly evaluation was mortality greater than 20% in the control group. For mortality rates between 5 and 20%, the Abbott correction was applied [16]. Statistical analysis used the Shapiro–Wilk W-test followed by the Mann–Whitney–Wilcoxon test, both in the RStudio version 1.2.5001 program [17].

## Results

**Fig. 2:**
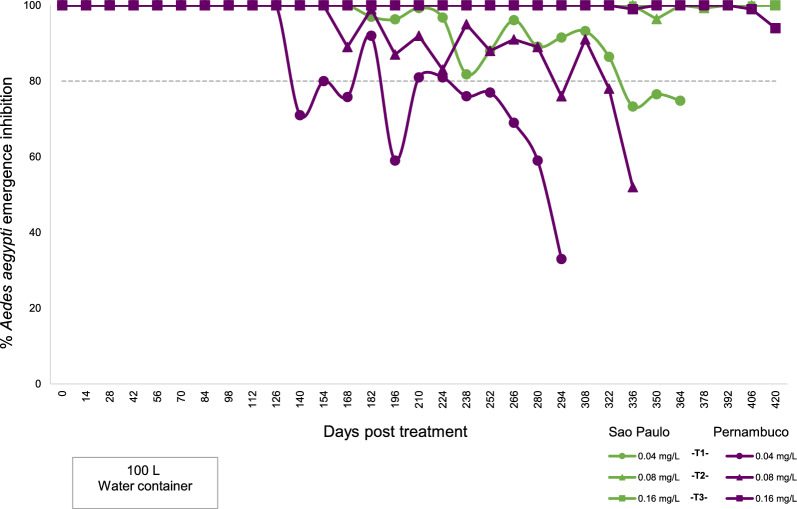
*Aedes aegypti* emergence inhibition rate in São Paulo and Pernambuco in water containers treated with SumiLarv^®^ 2MR at different concentrations (arrow = day 0 treatment). Containers with 100 l and 0.04 mg/l (test 1), 0.08 mg/l (test 2), and 0.16 mg/l (test 3) concentrations. Bioassay was carried out in semi-field conditions in Brazil from 2017 to 2019

A total of 31 assessments were recorded for 420 days, with a total of 71,700 larvae of *Ae. aegypti* exposed to the SumiLarv^®^ 2MR product. The persistence of the product in Pernambuco was 238 days for a concentration of 0.04 mg/l, 322 days for a concentration of 0.08 mg/l, and 420 days for a concentration of 0.16 mg/l in 100-l containers. The tests conducted in São Paulo showed higher persistence (322 days) for 0.04 mg/l, and the other concentrations of the product, 0.08 and 0.16 mg/l, maitained an emergence inhibition rate above 80% during the entire evaluated period (Fig. 2). In total, three assessments were excluded from the study because of a mortality rate above 20% in the control group (Additional file 1).

**Fig. 3:**
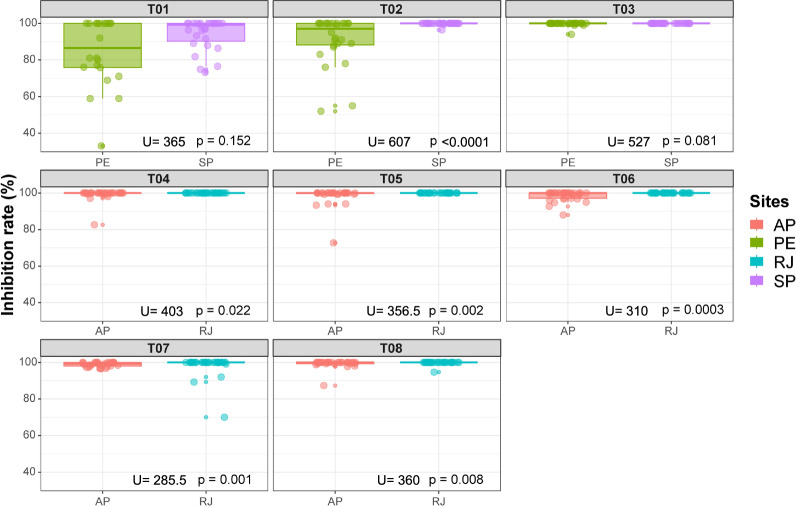
Nonparametric analysis using the Wilcoxon rank-sum test to assess the residual variability of SumiLarv^®^ 2MR in different *Aedes aegypti* populations. Brazilian states: *PE* Pernambuco, *SP* São Paulo, *AP* Amapá, *RJ* Rio de Janeiro

Statistical analyses showed a significant difference between the residual activity of the product from the populations of São Paulo and Pernambuco in the 0.08-mg/l concentration, which corresponds to test 2 of the bioassay (Fig. 3).

**Fig. 4:**
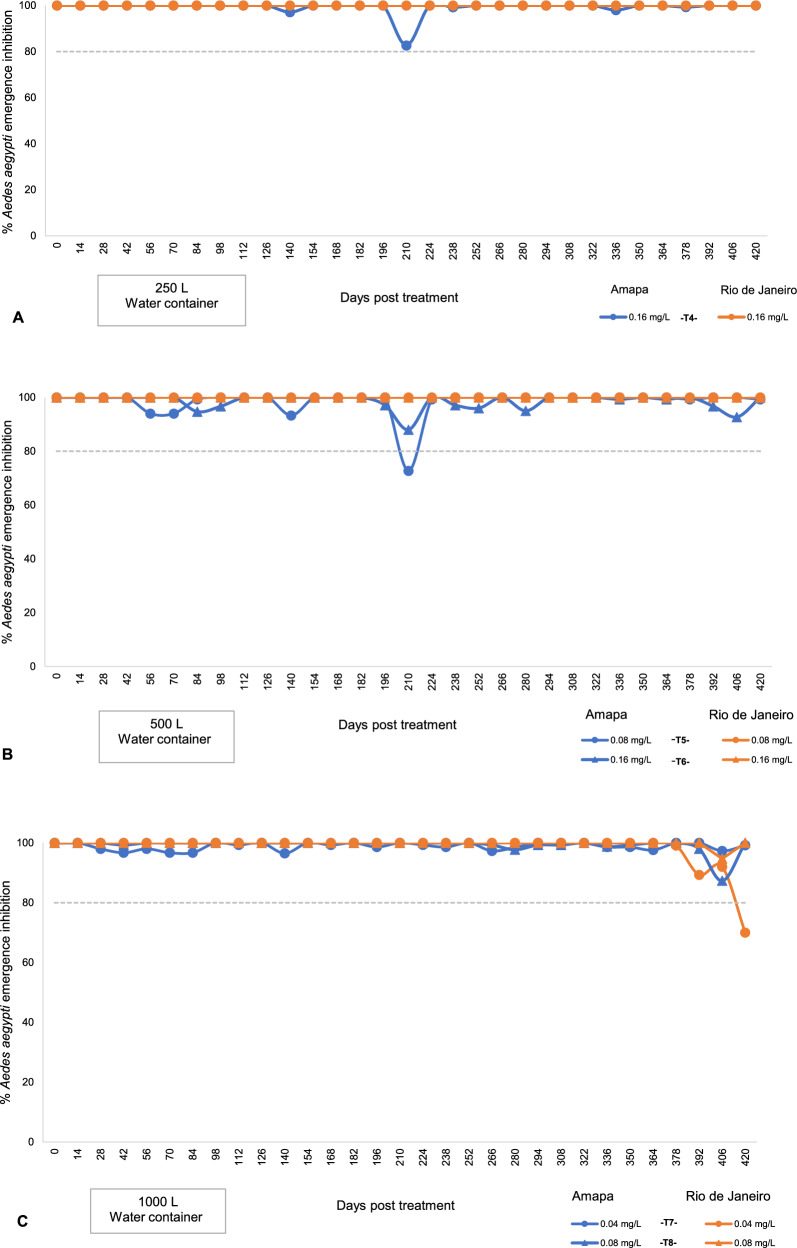
*Aedes aegypti* emergence inhibition rate of Amapá and Rio de Janeiro in water containers treated with SumiLarv^®^ 2MR (arrow = day 0 treatment) in different concentrations. **A** Containers with 250 l (test 4); **B** containers with 500 l; **C** containers with 1000 l. Bioassay carried out in a semi-field condition in Brazil from 2017 to 2019

In Amapá and Rio de Janeiro, tests in 250-, 500-, and 1000-l water storage tanks showed residual activity for 420 days (Fig. 4). Two assessments were excluded from the analysis from test 4 and four from tests 5–8 because of a mortality rate above 20% in the control group (Additional file 1). Statistical analyses also showed that there was a significant difference (*P* < 0.05) between the emergence inhibition rate in all tested populations of *Ae. aegypti* from Amapá and Rio de Janeiro (Fig. 3).

Across the four regions, the control group mortality rate was higher than expected in the first assessment (Additional file 1).

**Fig. 5:**
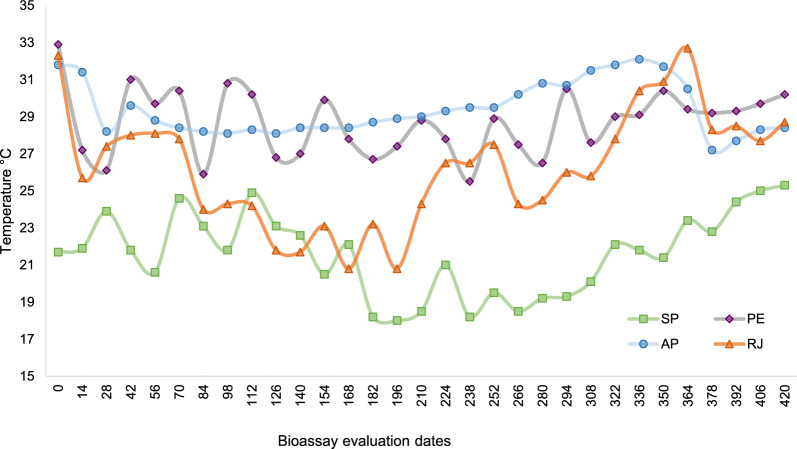
Water temperature variation observed in different containers treated with SumiLarv^®^ 2MR during semi-field tests in four Brazilian states: São Paulo (SP), Pernambuco (PE), Amapá (AP), and Rio de Janeiro (RJ)

The replacement of 60% of the water per week in each container used to simulate regular domestic water consumption showed that the SumiLarv^®^ 2MR remained effective during the assessments. Throughout the bioassay, the water temperature ranged between 18.0 and 25.3 °C, with pH ranging from 5.9 to 7.7 in the state of São Paulo; 25.5–32.9 °C and pH 6.5–7.6 in Pernambuco; 27.2–32.1 °C and pH 4.4–5.8 in Amapá; and 20.8–32.7 °C and pH 5.0–7.1 in Rio de Janeiro (Fig. 5).

## Discussion

Water storage tanks are very common as a domestic container in Brazil, and the weekly dynamics in the use of water, as well as specific environmental conditions, can interfere in the residual activity of products aimed to control *Ae. aegypti* [9, 18–20]. For this reason, the performance of multicentric bioassays can indicate the effectiveness of products considering the actual conditions of each location, since differences among regions can be seen in these parameters and others such as temperature and water pH.

The great territorial extent of Brazil contributes to temperature variations between the different regions of the country. Thus, the temperatures in the southeast, north, and northeast are different, but high temperatures are observed in all places throughout the year. A study investigating Amazon simulated climate change scenarios showed accelerated *Ae*. *aegypti* larval development in the case of increased global temperature [21]. The approach carried out in our study indicated important differences in water temperature when comparing semi-field conditions. The maximum water temperature in São Paulo was around 25 °C, while in Pernambuco this was the minimum temperature observed. A difference of 6.4 °C was found when comparing the average minimum water temperature in Amapá (27.2 °C) and Rio de Janeiro (20.8 °C), while the maximum temperature in both locations was around 32 °C. Statistical analyses showed that there was a significant difference between them.

Temperature plays an important role in *Ae. aegypti* development [22]; the temperature for the rearing of larvae for all four populations averaged 26 ± 2 °C in the insectary environment. The larvae were added to water storage tanks in semi-field conditions in the third stage, and one of the reasons for mortality above 20% in the control group may have been the variations in temperature between the laboratory environment and the semi-field conditions. Assessment results that could not be adjusted by Abbott’s formula were excluded from the analyses.

Smaller containers showed lower residual activity than larger containers, for both high and low temperature averages, in both Pernambuco and São Paulo. As for Amapá and Rio de Janeiro, larger tanks were used, and the emergence inhibition rate was above 80%, with significant differences observed between groups. Variations in the emergence inhibition rate for 100-l storage tanks may be associated with variations in temperature. The question remains whether temperature variation was indeed responsible for the residual activity difference in containers with less water, or if the lower concentration of the product influenced the outcome. Further studies are needed to provide conclusive answers to these questions.

Another important abiotic factor for larval development is the water pH [23], which was measured during the assessments, and did not show a significant difference between São Paulo, Pernambuco, and Rio de Janeiro (range 6–7.5). However, in Amapá, the water pH was considered acidic throughout the study period, ranging from 4.4 to 5.8. This is because the water in Amapá did not go through a treatment plant, as in the other places of this study, and was taken directly from the well. We believe that the presence of chlorine in water treatment may have contributed to the larval mortality in the control group during the first evaluation of assessments in São Paulo, Rio de Janeiro, and Pernambuco.

Abiotic factors such as temperature and pH can also influence the residual activity of other products, such as *Bacillus thuringiensis* var. *israelensis* and Temephos^®^, whose results in semi-field and field conditions were important for decision-making in mosquito control in Brazil [18]. In our study, SumiLarv^®^ 2MR showed inhibition of adult emergence for more than 60 days in all tests.

According to the national *Aedes* control program, visits to properties for breeding site elimination and treatment of unmoved containers must take place every 2 months. Thus, the ideal setting is using the product as a control measure with a minimum residual effect of 60 days. SumiLarv^®^ 2MR showed persistence greater than 400 days in 250-, 500-, and 1000-l water storage tanks in Amapá and Rio de Janeiro, and in 100-l tanks in Pernambuco and São Paulo at a concentration of 0.16 mg/l. Further consideration should be given to the fact that resins in São Paulo and Pernambuco were reduced before they were added to containers, confirming the homogeneity of the activity and the efficiency of the product in lower water volume.

The long-term residual activity of SumiLarv^®^ 2MR observed in the tests was related to the slow release of PPF in water, since the juvenile hormone analogue influences larval development and inhibits adult emergence. Field studies performed in Cambodia using SumiLarv^®^ 2MR in field conditions showed good acceptance by the population because the PPF did not cause a bad odor in the water, and because of its long-term effectiveness when compared to other larvicides. Health volunteer engagement was essential in the process of explaining the products’ action, leading to higher acceptance by the population [11].

Previous studies with SumiLarv^®^ 2MR indicated residual efficacy for 6 months for domestic use simulation [24, 25]; however, the results presented in our study showed that the residual efficacy was greater than 1 year. In field studies conducted in Asian countries with the same product in similar water storage tanks, the SumiLarv^®^ 2MR discs were lost, demonstrating that an integrated approach combined with COMBI strategies is required for dengue control [11, 26, 27]. In studies where the product was added in containers with large amounts of organic matter, the results were also positive; according to the authors, organic matter in the water served as a reservoir of PPF [28].

SumiLarv in other formulations, such as in granules [29], plays an important role in control measures against mosquito-borne arbovirus. Dissemination stations using PPF have shown positive control results [30]. However, with the use of juvenile hormone analogues as routine procedures in different vector control programs, the factors of susceptibility and resistance to insecticides must be considered; in the USA, larvae of *Ae. aegypti* showed moderate resistance to PPF [31].

The PPF powder formulation, Sumilarv^®^ 0.5G, was used in Brazil’s National *Aedes* Control Program from 2010 to 2020. Rotation of larvicide classes is recommended [32] in order to guarantee the effectiveness of the insecticides over time [33] and to prevent selection for resistance, as demonstrated by several studies carried out in Brazil [34]. Our results showed residual activity greater than 400 days, indicating that SumiLarv^®^ 2MR can be useful in the current context of *Ae. aegypti* and *Aedes albopictus* dissemination. Similar results have been highlighted in studies combining multidisciplinary approaches [35, 36].

## Conclusions

The larval and pupal control performance of SumiLarv^®^ 2MR demonstrated in this multicenter semi-field study suggests that this new PPF formulation is a promising alternative for *Aedes* control. Further studies are needed in field conditions incorporating active community participation as an essential strategy for improving acceptance of the product and the success and sustainability of its implementation in *Aedes* control programs.

### Supplementary Information

**Additional file 1.** Mortality rate above 20% in the control group (number 1) excluded from the statistical residuality study, during SumiLarv^®^ 2MR evaluation in four populations of *Aedes aegypti* mosquitoes in different Brazilian regions. 0  < 20% in the control group; 1  ≥ 20% in the control group. *T* test.

## Data Availability

All data supporting the findings of this study are available within the paper and its associated file.

## Abbreviations

IVM: Integrated vector management
WHO: World Health Organization
PPF: Pyriproxyfen
PNCD: Brazilian Dengue Control Program
IGR: Insect growth regulator
COMBI: Communication for behavioral impact
IAM: Aggeu Magalhães Institute
IEPA: Institute for Scientific and Technological Research of the State of Amapá
LBCVIV: Laboratory of Biology, Control and Surveillance of Vector Insects
IOC: Oswaldo Cruz Institute
FIOCRUZ: Oswaldo Cruz Foundation
SUCEN: Superintendency for the Control of Endemic Diseases
