# Supplementary material for: Impact of SumiLarv® 2MR on Aedes aegypti larvae: a multicenter study in Brazil
Source: Parasit Vectors. 2024 Feb 26;17:88. doi: 10.1186/s13071-023-06064-w (PMC10895835; doi:10.1186/s13071-023-06064-w)
Supplement: Supplementary file 1 — Additional file 1. Mortality rate above 20% in the control group (number 1) excluded from the statistical residuality study, during SumiLarv® 2MR evaluation in four populations of Aedes aegypti mosquitoes in different Brazilian regions. 0 < 20% in the control group; 1 ≥ 20% in the control group. T test. [file 13071_2023_6064_MOESM1_ESM.docx]

**Additional file 1 –**

| **Days post tratead** | **Sao Paulo and Pernambuco** | | |  |  | **Amapa and Rio de Janeiro** | | | | | | |
| --- | --- | --- | --- | --- | --- | --- | --- | --- | --- | --- | --- | --- |
|  | **T1** | **T2** | **T3** |  |  | **T4** |  | **T5** | **T6** |  | **T7** | **T8** |
| **0** | 1 | | |  |  | 1 |  | 1 | |  | 1 | |
| **14** | 0 | | |  |  | 0 |  | 0 | |  | 0 | |
| **28** | 1 | | |  |  | 0 |  | 0 | |  | 0 | |
| **42** | 0 | | |  |  | 0 |  | 0 | |  | 1 | |
| **56** | 0 | | |  |  | 0 |  | 0 | |  | 0 | |
| **70** | 0 | | |  |  | 0 |  | 0 | |  | 0 | |
| **84** | 0 | | |  |  | 0 |  | 0 | |  | 0 | |
| **98** | 0 | | |  |  | 0 |  | 0 | |  | 0 | |
| **112** | 0 | | |  |  | 0 |  | 0 | |  | 0 | |
| **126** | 0 | | |  |  | 0 |  | 0 | |  | 0 | |
| **140** | 0 | | |  |  | 0 |  | 0 | |  | 0 | |
| **154** | 0 | | |  |  | 0 |  | 0 | |  | 0 | |
| **168** | 0 | | |  |  | 0 |  | 0 | |  | 0 | |
| **182** | 0 | | |  |  | 0 |  | 0 | |  | 0 | |
| **196** | 0 | | |  |  | 0 |  | 0 | |  | 0 | |
| **210** | 0 | | |  |  | 0 |  | 1 | |  | 1 | |
| **224** | 0 | | |  |  | 0 |  | 0 | |  | 0 | |
| **238** | 0 | | |  |  | 0 |  | 0 | |  | 0 | |
| **252** | 0 | | |  |  | 0 |  | 0 | |  | 0 | |
| **266** | 1 | | |  |  | 0 |  | 0 | |  | 0 | |
| **280** | 0 | | |  |  | 0 |  | 0 | |  | 0 | |
| **294** | 0 | | |  |  | 0 |  | 0 | |  | 0 | |
| **308** | 0 | | |  |  | 0 |  | 0 | |  | 0 | |
| **322** | 0 | | |  |  | 0 |  | 0 | |  | 0 | |
| **336** | 0 | | |  |  | 0 |  | 0 | |  | 0 | |
| **350** | 0 | | |  |  | 0 |  | 0 | |  | 0 | |
| **364** | 0 | | |  |  | 0 |  | 1 | |  | 1 | |
| **378** | 0 | | |  |  | 1 |  | 1 | |  | 0 | |
| **392** | 0 | | |  |  | 0 |  | 0 | |  | 0 | |
| **406** | 0 | | |  |  | 0 |  | 0 | |  | 0 | |
| **420** | 0 | | |  |  | 0 |  | 0 | |  | 0 | |
